# Effect of Heavy Atoms on the Thermal Stability of α-Amylase from *Aspergillus oryzae*

**DOI:** 10.1371/journal.pone.0057432

**Published:** 2013-02-25

**Authors:** Michihiro Sugahara, Michiyo Takehira, Katsuhide Yutani

**Affiliations:** RIKEN SPring-8 Center, Sayo, Hyogo, Japan; Weizmann Institute of Science, Israel

## Abstract

Currently, there are no versatile and established methods for improving stability of proteins. In an entirely different approach from conventional techniques such as mutagenesis, we attempted to enhance enzyme stability of α-amylase from *Aspergillus oryzae* using a heavy-atom derivatization technique. We evaluated changes in stability using differential scanning calorimetry (DSC). Candidate heavy atoms were identified using the Heavy-Atom Database System *HATODAS*, a Web-based tool designed to assist in heavy-atom derivatization of proteins for X-ray crystallography. The denaturation temperature of α-amylase derivatized with gadolinium (Gd) or samarium (Sm) ions increased by 6.2 or 5.7°C, respectively, compared to that of the native protein (60.6°C). The binding of six Gd ions was confirmed by X-ray crystallography of the enzyme at 1.5 Å resolution. DSC and dynamic light-scattering data revealed a correlation between stability and the aggregation state upon addition of Gd ions. These results show that *HATODAS* search is an effective tool for selecting heavy atoms for stabilization of this protein.

## Introduction

Enzymes are currently used in many different industrial products and processes [1]. To enhance thermal stability of highly useful enzymes for industrial applications, protein-engineering techniques such as site-directed mutagenesis, random mutagenesis, recombination, and directed-evolution techniques have been successfully employed for various proteins [2], [3]. To date, most stability improvements have been based on the introduction of point mutations into proteins. In general, however, there are no established methods that can be used to predict the effect of mutations on thermal stability, because even single amino-acid substitutions affect various stabilization factors depending on the surrounding environment, substituted residues, and structural changes due to the mutations [4]–[6]. Therefore, a versatile and simple method for thermal stabilization of enzymes is required.

As well as the site directed mutagenesis, techniques for protein stability by using additives such as covalent cross-linkers and ligands have been well studied since early times. The covalent cross-linking technique by bifunctional chemical modification reagents [7], [8] as an additive has been successfully used to enhance the stability of proteins [9]. The cross-linking in proteins reduces the configurational backbone chain entropy of the unfolded polypeptide, resulting in increase in conformational stability [10]–[12]. On the other hand, the binding of ligand increases the stability of proteins in response to Schellman's binding theory [13], [14]. The stabilization mechanism can be explained stoichiometrically by the shift of the folding-unfolding equilibrium toward the folded state caused by the higher affinity of ligand to the folded state [15]. Thus these techniques using the additives have the advantages of being simple and quick, and are useful for enhancing protein stability efficiently.

Metal ions that bind with high affinity to specific sites often stabilize the conformation of proteins [16]–[21]. In protein X-ray crystallography, experimental phasing from heavy atom–derivatized crystals is a major technique in structure determination [22]. The identification of heavy atoms that bind proteins can be useful both for improving crystallization success rates and in some cases for enhancing the thermal stability of these proteins [23]. However, determination of candidate heavy atoms is laborious and time-consuming. Recently, to facilitate the heavy-atom derivatization process, we developed the Heavy-Atom Database System (*HATODAS*, http://hatodas.harima.riken.jp), which suggests candidate heavy-atom reagents for a target protein based on a search against a database of known heavy atom–liganded proteins, using the amino-acid sequence and crystallization conditions as the query terms [24], [25]. We believe that *HATODAS* search could also contribute to the efficient selection of candidate heavy atoms for use in protein stabilization using the derivatization technique.

Here we present such a method, which utilizes derivatization with heavy atoms identified through *HATODAS* search. This study was designed to investigate whether there is a relationship between heavy-atom-derivatized protein and its conformational stability; to date, there has been no detailed study of the effect of heavy atoms on protein stability. To elucidate thermal behavior of a derivatized protein, we used differential scanning calorimetry (DSC) to investigate the stability of *Aspergillus oryzae* α-amylase (Ao α-amylase) containing heavy atoms. This enzyme catalyzes the hydrolysis of the α-1,4 glycosidic linkages in raw and soluble starches, and is used in numerous industrial applications for starch conversion in value-added products. The stability of Ao α-amylase containing heavy atoms was evaluated over a wide range of concentrations by heat denaturation, and the structures of the derivatized proteins were determined by X-ray crystallography. We also discuss the thermostabilization mechanism of heavy atom–derivatized Ao α-amylase.

## Results and Discussion

### Heavy-atom selection for thermostabilization

Six candidate heavy atoms for thermostabilization of Ao α-amylase were selected using the program *HATODAS*. The order of the top six was (1) mercury (Hg), (2) bromine (Br), (3) platinum (Pt), (4) samarium (Sm), (5) lead (Pb), and (6) thallium (Tl).

Because heavy atoms sometimes act as inhibitors, we first investigated whether derivatized proteins retained their enzymatic activity. We examined the activity of Ao α-amylase with each heavy atom at room temperature; digestion of starch was assayed by monitoring the disappearance of the starch-iodine color. In the presence of the six heavy atoms, activity was preserved, indicating that none of these heavy atoms is a potent inhibitor of the enzyme. In general, heavy atoms bind to characteristic residues: Hg, Au, and Ag tend to bind to Cys or His; Pt binds to Met or His; and Pb and lanthanides bind to Asp or Glu [25]. To avoid inactivation of the enzyme, heavy atoms should be selected based on the types of amino acids constituting the protein active site.

To further characterize these heavy atoms, we examined the stability of Ao α-amylase derivatized with each candidate. Solutions of each derivatized Ao α-amylase were incubated in a water bath at ∼70°C for 5 min, and the remaining activity was evaluated at room temperature. Only Sm, the fourth of six candidate atoms in terms of stabilization potential, had a significant effect on thermal stability of the enzyme; the other samples did not exhibit enzymatic activity after heat treatment. Although we confirmed that the Ao α-amylase crystals had been successfully derivatized with Hg ion, which *HATODAS* predicted would yield the greatest stabilization, this ion did not in fact stabilize this protein. In any case, use of Hg ions should be avoided because the accumulation of Hg from industrial uses poses serious environmental problems.

Based on these findings, we excluded heavy atoms other than Sm from further characterization, since they did not enhance the stability of this enzyme. However, because structure determination of the Sm-binding protein was not successful (see Materials and Methods), we performed additional *HATODAS* searches for lanthanide ions as a substitute for Sm, resulting in the identification of gadolinium (Gd) as a new candidate. Activity evaluation after heat treatment revealed that derivatization with Gd ion yielded heat-stabilization similar to that observed with Sm ion. Furthermore, we were able to successfully determine the crystal structure of Gd-derivatized protein. Therefore, we elected to further characterize the Gd-derivatized Ao α-amylase.

### Differential scanning calorimetry of Ao α-amylase

**Figure 1:**
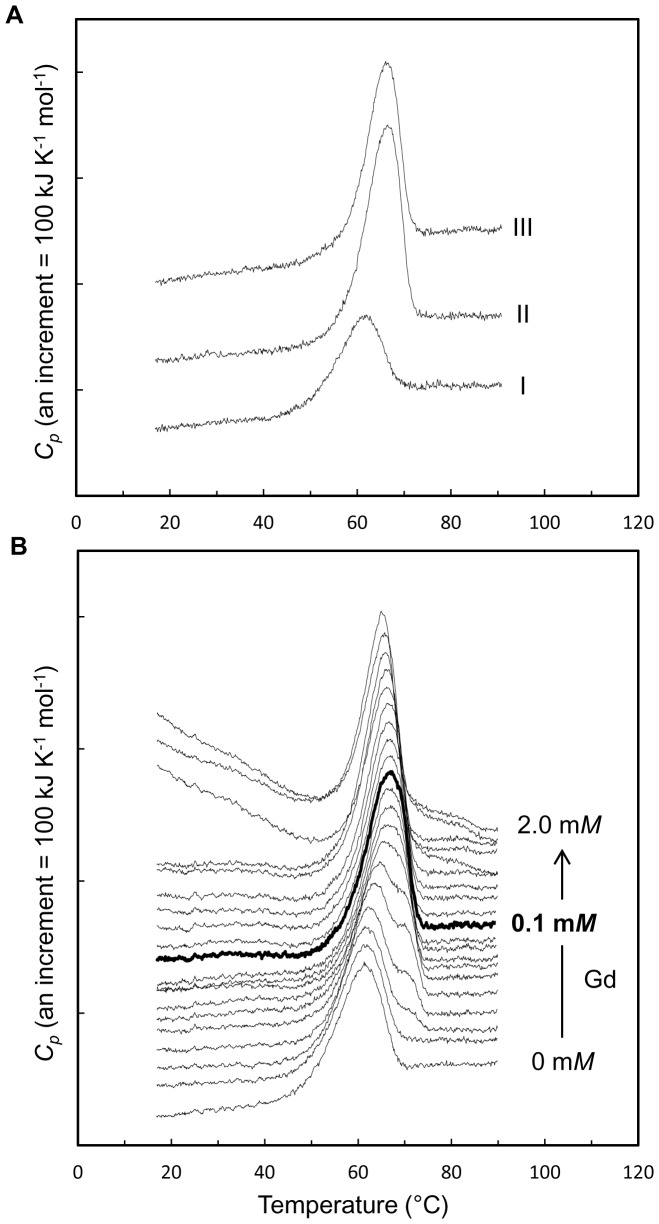
DSC curves of Ao α-amylase with heavy atoms at pH 5.8. (A) 0.2 mg/mL Ao α-amylase with heavy atoms. (I) no heavy atom, (II) 0.2 m*M* GdCl_3_, (III) 0.2 m*M* SmCl_3_. (B) Gd concentration dependence of the denaturation temperature of 0.4 mg/mL Ao α-amylase with 0–2.0 m*M* GdCl_3_ (0, 0.01, 0.02, 0.03, 0.04, 0.05, 0.06, 0.07, 0.08, 0.09, 0.10, 0.11, 0.12, 0.15, 0.2, 0.3, 0.5, 0.9, 1.0, and 2.0 m*M*). A concentration of 0.1 m*M* GdCl_3_ gave the highest *T*_d_ value, 67.3°C (heavy line). The denaturation temperature, *T*_d_, represents the temperature corresponding to the peak of the DSC curve observed at a scan rate of 200°C/hour.

Based on the initial findings regarding enzymatic activity after heat treatment, we selected two heavy atoms, Gd and Sm, for further study of thermal stabilization of Ao α-amylase. Enzymes derivatized with GdCl_3_ or SmCl_3_ were examined by DSC to detect differences in their thermal behavior (Fig. 1A). In the absence of heavy atoms, the denaturation temperature (*T*_d_) for Ao α-amylase was 60.6°C. On the other hand, *T*_d_ increased to 66.8°C or 66.3°C, respectively, when a 0.2 mg/mL solution of Ao α-amylase was incubated with 0.2 m*M* of either GdCl_3_ or SmCl_3_. Thus, the *T*_d_ of Ao α-amylase increased by ∼6°C in the presence of these heavy atoms, indicating that the stability of the protein was remarkably improved by both ions.

We next conducted DSC experiments to evaluate the changes in stability under different concentrations (0–2.0 m*M*) of GdCl_3_, using a 0.4 mg/mL solution of Ao α-amylase (Fig. 1B, Table 1). The *T*_d_ increased with increasing Gd concentration between 0 and 0.1 m*M* GdCl_3_. At 0.1 m*M* GdCl_3_, Ao α-amylase exhibited the highest *T*_d_ value, 67.3°C; this value is 6.3°C higher than that of the native protein. The DSC curves show that *T*_d_ declined with increasing concentration from 0.11 to 2.0 m*M* GdCl_3_, due to protein aggregation, as described below (Table 1). In the DSC measurements of the cooling and reheating processes, no excess heat capacity curve was observed in the presence or absence of Gd ions, suggesting that the heat denaturation of Ao α-amylase was irreversible under the conditions of the experiment.

**Table 1:** Results of DSC and DLS experiments for 0.4 mg/mL Ao α-amylase in the presence of Gd ions at pH 5.8.

		DSC	DLS			
			peak 1		peak 2	
GdCl_3_	mole ratio	*T*_d_	molecular mass*	mass	molecular mass*	mass
m*M*	protein: Gd	°C	kDa	%	kDa	%
0	1∶0	61.2	60.3	100		0
0.01	1∶1.3	61.0	53.5	100		0
0.02	1∶2.6	61.6	60.1	99.8	7099	0.2
0.03	1∶3.9	62.4	51.4	99.5	5882	0.5
0.04	1∶5.3	63.7	43.2	99.3	2890	0.7
0.05	1∶6.6	64.3	40.6	98.8	6582	1.2
0.06	1∶7.9	65.6	35.0	99.4	9821	0.6
0.07	1∶9.2	66.6	45.6	98.9	9588	1.1
0.08	1∶11	66.9	56.2	98.9	2.3×10^4^	1.1
0.09	1∶12	66.9	40.9	99.2	2.6×10^4^	0.8
0.10	1∶13	67.3	42.8	99.3	3.3×10^4^	0.7
0.11	1∶15	66.6	46.0	98.6	4.2×10^4^	1.4
0.12	1∶16	66.8		0	3.6×10^4^	100
0.15	1∶20	66.7		0	5.0×10^4^	100
0.20	1∶26	66.9		0	1.8×10^5^	100
0.30	1∶40	66.4		0	3.0×10^5^	100
0.50	1∶66	66.2		0	10^6^	100
0.90	1∶118	65.9		0	10^6^	100
1.00	1∶132	65.6		0	10^6^	100
2.00	1∶263	65.1		0	10^6^	100

* molecular mass estimated from the measured radius (*DYNAMICS*, Protein Solutions).

A maximum *T*_d_ is observed at a concentration of 0.1 m*M* GdCl_3_ (bold).

### Crystal structure of Gd derivatized Ao α-amylase

**Figure 2:**
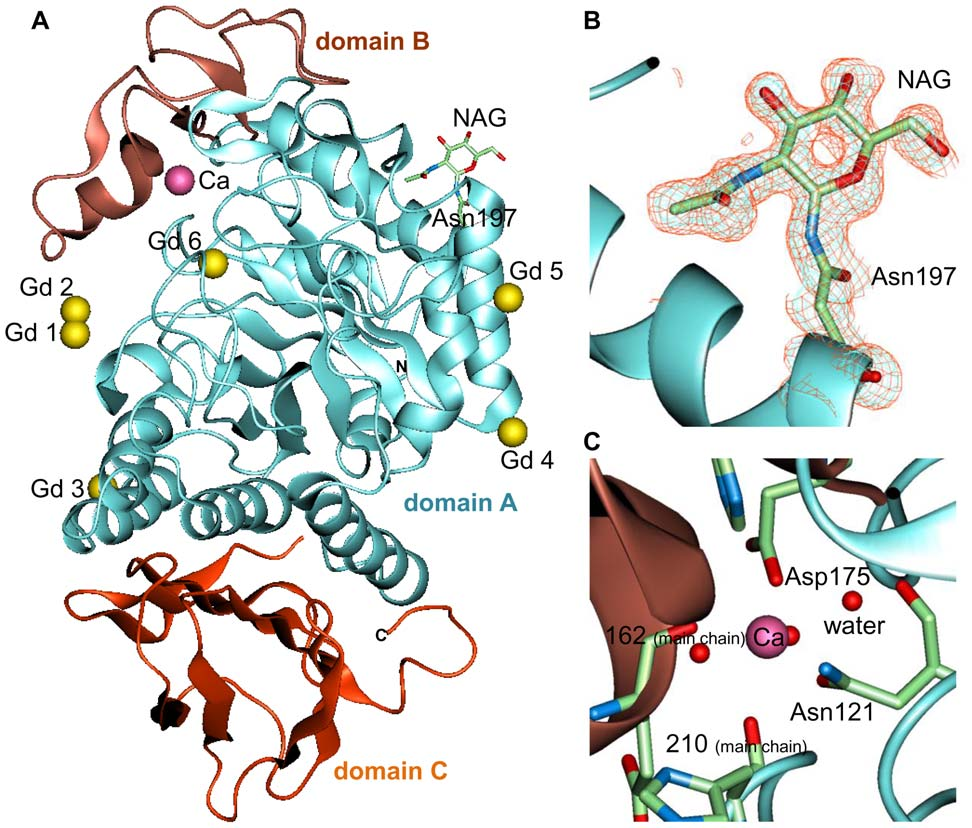
The crystal structure of Gd-derivatized Ao α-amylase. (A) Overall structure shown in ribbon diagram. The three domains, A, B, and C, are colored light blue, brown, and orange, respectively. NAG molecule and residue Asn197 are depicted as licorice models. Bound Ca and Gd ions are depicted as pink and yellow spheres, respectively. Close-up view of (B) NAG binding site with (2*F*o–*F*c) electron-density map contoured at 1.2*σ* (blue mesh) and 0.6*σ* (orange mesh) level and (C) Ca binding site. The residues are depicted as licorice models. The perspective is the same as that in Fig. 2A. Drawn in *QUANTA2000*.

To elucidate the stabilization mechanism of Ao α-amylase with a *T*_d_ of 67.3°C, we determined the structures of native and Gd-derivatized proteins by X-ray crystallography. The crystal structure of the Gd-derivatized protein is shown in Fig. 2A. The structure consists of three domains: a central main domain, A (residues 1–121 and 177–380), formed by an N-terminal catalytic (β/α)_8_-barrel; a small β-pleated domain, B (122–176), protruding between β3 and α3; and a C-terminal globular domain, C (residues 381–478), consisting of a β-structure with a Greek-key motif [26]–[32]. This structure is similar to previously reported X-ray structures of Ao α-amylase.

N-acetyl glucosamine (NAG)– and calcium-binding sites were present in the crystal structures of both the native and the derivatized protein (Fig. 2B and 2C). The positions of these two ligands are identical to those in the reported structure of α-amylase (PDB code: 2gvy) [32]. Since no NAG was added during the crystallization of Ao α-amylase, this endogenous NAG may have been incorporated during the preparation steps. A calcium-binding site is located at the interface between the catalytic A domain and the B domain (Fig. 2C). This calcium ion is recognized by interactions with the side-chains of Asn121 and Asp175, the main-chain atoms of residues Glu162 and His210, and three water molecules. A characteristic feature of α-amylases is their requirement for calcium ions for activity and conformational stability [33].

**Figure 3:**
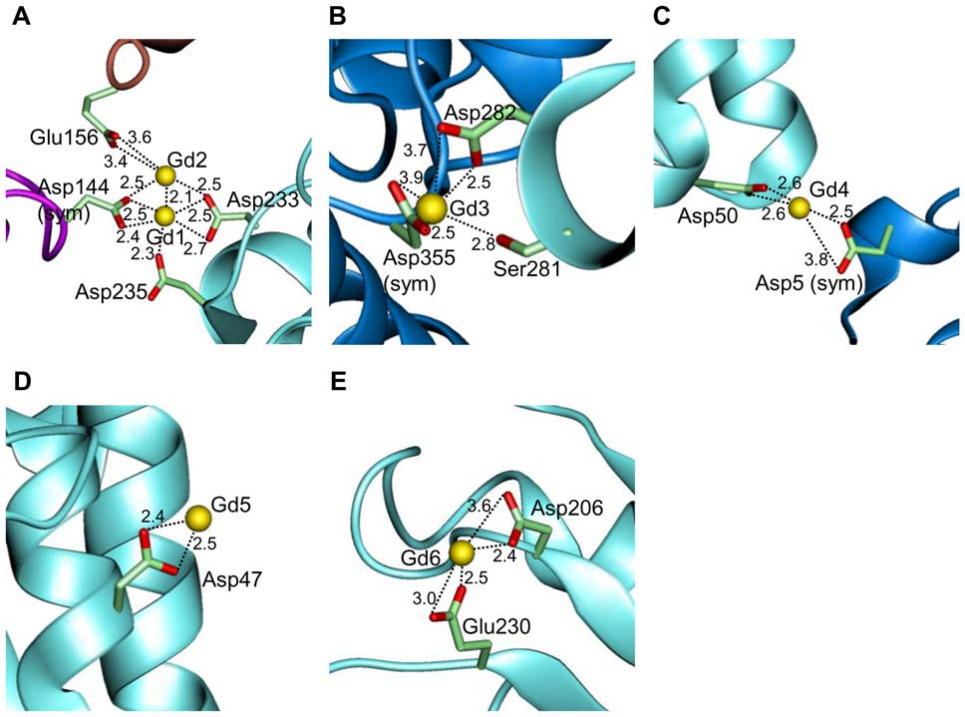
Close-up view of Gd binding sites. (A–E) Bound Gd ions and the interacting residues are depicted as yellow spheres and licorice models, respectively. The neighboring symmetry-related chains of domain A and B are colored blue and purple, respectively. The perspective is the same as that in Fig. 2A. Drawn in *QUANTA2000*.

In the derivatized protein structure, we found six bound Gd ions (Fig. 2A). The Gd1, Gd2, Gd3, and Gd4 ions are located in between the residues of the asymmetric chain and those of a neighboring symmetry-related chain (Fig. 3A–C). The Gd1–4 ions may act as an interchain linker, resulting in oligomerization of Ao α-amylase molecules. Gd1, Gd3, and Gd4 ions exclusively mediate interactions between the proteins, as opposed to within a single protein molecule. However, the interactions involving Gd1, Gd3, and Gd4 make no contribution to stability, because dynamic light-scattering analysis of the stabilized protein in solution suggested that it exists in a monomeric state (see below and Table 1). The position of the Gd2 ion, which interacts with both Asp233 of domain A and Glu156 of domain B, suggests that Gd2 contributes to the conformational stability of Ao α-amylase (Fig. 3A). The Gd5 ion is bound to Asp47 on the molecular surface (Fig. 3D). One Gd-binding site, Gd6, was present on the catalytic pocket of domain A (Fig. 3E). The binding of Gd6 ion to Asp206 and Glu230 was weak, with a site occupancy of 0.2. Thus, the derivatized enzyme may retain activity because of the low occupancy of the Gd6 site.

To confirm the structural changes due to derivatization, we performed a C^α^ superposition analysis using the program *LSQKAB* [34]. The C^α^ r.m.s.d. (root mean square deviation) between the derivative and native Ao α-amylase structures was 0.26 Å, indicating that the overall crystal structure of the derivatized protein was highly similar to the native structure. In addition, to obtain additional information regarding structural changes in solution with Gd ions, we generated near-UV circular dichroism (CD) spectra in the ranges from 250 to 320 nm (data not shown). The CD spectrum of Ao α-amylase with Gd ions was indistinguishable from that of the protein without Gd ions, further supporting that the tertiary structure of Gd-derivatized Ao α-amylase is similar to that of the native protein. From these results, we conclude that the Gd ions do not affect the overall conformation of the Ao α-amylase molecule.

In this study, it still remains unclear whether the Gd2 ion plays a significant role in stabilizing the Ao α-amylase structure. It might be difficult to specify effects of Gd ions by site-directed mutagenesis because an introduction of the point mutation in proteins affects not only the stabilizing factors of the mutation site but also those of other parts far from the substitution site [4]–[6]. The stabilization of Ao α-amylase was mediated by Gd ions. The ligand binding is important to the increase in thermal stability in solution [13], [14]. In a study for thermal stability of tryptophan synthase, Ogasahara *et al*. have shown that the simulated interaction between the subunits with the order of 10^8^ *M* ^−1^ of binding constant remarkably enhances the thermal stability of the protein without conformational change [15]. The increase in *T*_d_ of Gd-binding Ao α-amylase without conformational change could be caused by the increase in the binding constant. On the other hand, covalent cross-linking in proteins by disulfide or chemical cross-linkers restrains the folding and unfolding of the protein, resulting in a decrease in the conformational entropy of the unfolded polypeptide [10]–[12]. Therefore, the stabilization mechanism of the Gd-binding protein is completely different from that of the cross-linked protein. Our technique using heavy atoms is one of those methods for thermal stabilization of proteins, suggesting that the application of a variety of techniques is essential to eliminate the time consuming procedure during the trial and error process.

### Dynamic light-scattering (DLS) experiment

A DLS experiment revealed that Ao α-amylase exists in a monomeric state in the absence of Gd ions (Table 1). However, the monomeric state shifted toward the oligomeric state upon addition of Gd ions. For GdCl_3_ concentrations between 0.02 and 0.11 m*M*, bimodal peaks were observed (98.6–99.8% mass in the monomeric state and 0.2–1.4% mass in the oligomeric state). For Gd concentrations between 0.12 and 2.0 m*M*, a bimodal analysis revealed an estimated molecular mass of 10^4^–10^6^ kDa, suggesting aggregation of Ao α-amylase in solution. Thus, the inclusion of excess Gd ions actually appears to promote the aggregation of this enzyme.

At 0.1 m*M* GdCl_3_, i.e., the condition that confers maximum *T*_d_, 99% of the protein is in the monomeric state (Table 1), indicating that the Gd ions in the monomeric crystal structure (Fig. 3) contribute predominantly to the stabilization of this protein. The Gd ion concentrations associated with protein aggregation correspond to the concentrations at which the *T*_d_ decreases. The formation of aggregates might be due to Gd ions acting as inter-chain linkers (Gd1, 3, 4 in Fig. 3). Although the addition of excess Gd ions negatively affects the stability of this protein, the *T*_d_ in the aggregated state under in 2.0 m*M* GdCl_3_ is still approximately 4°C higher than that of the native protein. These results indicate that the stability of Ao α-amylase does not deteriorate significantly upon aggregation. In the crystal structure, one molecule of α-amylase binds to six Gd ions. However, the maximum *T*_d_ is observed at a 1∶13 molar ratio of protein to Gd (Table 1), implying that Gd ions interact with Ao α-amylase in the equilibrium state with weak binding constants.

In summary, we examined the applicability of heavy-atom derivatization as a stabilization technique, using Ao α-amylase protein in the presence of lanthanide ions. The *T*_d_ of Ao α-amylase increased in the presence of Gd ion, up to a maximum at 0.1 m*M* GdCl_3_, and decreased relative to this maximum at higher concentrations. At the maximum, the *T*_d_ value of the Gd-derivatized protein was 6°C higher than that of the native protein. The stabilization mechanism of the enzyme by Gd was elucidated by analysis of crystal structure solved at 1.5 Å resolution, and by observation of its physicochemical properties (such as changes in *T*_d_) and its oligomeric state in solution. The results suggest that the bound heavy atoms contribute substantially to the thermal stability of this enzyme. A similar stabilization by the heavy-atom derivatization technique, using ions selected by the *HATODAS* software, has been observed in a putative α-ribazole-5′-phosphate phosphatase from *Thermus thermophilus* HB8. The *T*_d_ of that protein in the presence of Pt ions was 95.6°C, 16.4 °C higher than that of the native protein (Sugahara *et al*., in preparation).

The results presented here confirm that *HATODAS* is a powerful tool for identifying heavy atoms for use in stabilization of two examined proteins, and suggest that the derivatization technique is useful for rapid and effective stabilization of proteins. One clear advantage of the heavy-atom derivatization technique is its simplicity: all that is required is the addition of heavy atoms to protein solutions. This method may, therefore, be applicable to a wide range of proteins.

## Materials and Methods

### Heavy-atom selection for thermostabilization

As a test protein for this work, we used commercially available α-amylase (endo-1,4-α-D-glucan glucohydrolase) from *Aspergillus oryzae* (EC 3.2.1.1, MW = 52.4×10^3^, Sigma, Cat. No. 10065) without further purification. The optimal conditions for α-amylase in industrial application are pH and no Ca^2+^ [35], [36]. The program *HATODAS* (http://hatodas.harima.riken.jp/) identified six candidate heavy atoms based on the amino-acid sequence of Ao α-amylase and pH 5.8 as the query terms. Ao α-amylase activity with the selected heavy atoms was examined using the starch-iodine assay method [37]. A 0.1 mL aliquot of Ao α-amylase at 0.4 mg/mL in 0.1 *M* MES-NaOH (pH 5.8) with 1.0 m*M* heavy atoms was incubated for 5 min at 70°C in a water bath; this temperature was chosen because *T*_d_ for Ao α-amylase is 61°C in the absence of heavy atoms. The reactions were initiated by adding 0.1 mL of 0.25% (*w/v*) starch solution as a substrate to α-amylase solutions at 20°C. The α-amylase activity was confirmed by adding 0.1 mL of iodine reagent [0.02% (*w/v*) I_2_, 0.2% (*w/v*) KI] to 0.2 mL of the protein-starch solution at 20°C.

### Differential scanning calorimetry (DSC)

DSC experiments were performed at scan rate of 200°C/hour using a VP-capillary DSC platform. For the measurements of Sm and Gd ions, the protein concentration was 0.2 mg/mL in 50 m*M* MES-NaOH (pH 5.8), containing 0.2 m*M* SmCl_3_ or 0.2 m*M* GdCl_3_. For the measurements of heavy-atom concentration dependence, the protein concentration was 0.4 mg/mL in 50 m*M* MES-NaOH buffer (pH 5.8) containing 0.01, 0.02, 0.03, 0.04, 0.05, 0.06, 0.07, 0.08, 0.09, 0.10, 0.11, 0.12, 0.15, 0.2, 0.3, 0.5, 0.9, 1.0, or 2.0 m*M* GdCl_3_. All samples were dialyzed overnight at 4°C against buffer without heavy atoms and then filtered through a membrane with 0.22 µm pores. DSC data were analyzed using the *Origin* software supplied with the instrument (MicroCal Inc.). Molar excess heat capacities (*C*_p_) were obtained by normalizing against the Ao α-amylase concentration and the volume of the calorimeter cell. Apparent denaturation temperature (*T*_d_) value was defined as the temperature associated with maximum *C*_p_.

### Crystallization and diffraction data collection

Diffraction-quality crystals of Ao α-amylase were obtained using the oil microbatch method [38] on the Autolabo automatic crystallization system [39]. A crystallization drop of 1.0 µL, in the presence of synthetic zeolite molecular sieves as heteroepitaxic nucleants [40], [41], was created by mixing 1∶1 mixture of 28.0 mg/mL protein solution in 0.02 *M* MES-NaOH (pH 5.8) and precipitant solution composed of 40% (*w/v*) polyethylene glycol (PEG) 8,000, 0.2 *M* CaCl_2_, and 0.1 *M* MES-NaOH (pH 5.8) in a well of a Nunc HLA crystallization plate (Nalge Nunc International) which was then covered with 20 µL of paraffin oil. The resulting Ao α-amylase crystals were submitted to a heavy-atom derivatization experiment. The Gd derivative was prepared by soaking native crystals with 10 m*M* GdCl_3_, 40% (*w/v*) PEG 8,000, 0.2 *M* CaCl_2_, 0.1 *M* MES-NaOH (pH 5.8). Although the SmCl_3_ derivatization was attempted using concentrations in the range 0.1–10 m*M*, this treatment caused the crystals to crack, and structure determination was therefore impossible.

All crystals were directly mounted in a cryoloop from the crystallization drop and flash-cooled at 100 K in a nitrogen gas stream. Complete diffraction data sets were collected using an in-house Rigaku R-AXIS VII image-plate detector with Cu *Kα* radiation and a Rigaku R-AXIS V image-plate detector with synchrotron radiation at BL26B1 of SPring-8, Japan [42]. All data were processed using the program *HKL-2000* [43].

### Structure determination

Positioning of one Ao α-amylase molecule in the asymmetric unit was accomplished using the molecular-replacement method as implemented in the program *MOLREP* [44], based on the crystal structure deposited in the Protein Data Bank (PDB code 2taa). The structure of the Gd-bound protein was isomorphous to that of the native form and was determined by difference Fourier analysis using the corresponding model of the native structure. Manual model revision was performed using *QUANTA2000* software (Accelrys Inc.).

Bound ions were observed in the structure of the Gd-soaked crystal. Based on comparison of temperature factors to those of neighboring atoms, and from their coordination with ion-binding residues, the bound ions are most likely to be Gd ions from the heavy-atom soaking reagent. This interpretation is in agreement with the fact that strong signals are observed at the ion sites in the anomalous Fourier map at a wavelength of 1.000 Å, prepared using the program *FFT* in CCP4 suite [45].

The program *CNS* [46] was used for structure refinement and electron-density map calculation. Each cycle of refinement with bulk solvent and overall anisotropic B-factor corrections consisted of rigid-body refinement, simulated annealing incorporating the slow-cool protocol, positional refinement, and B-factor refinement (individual or group). Several cycles of model revision and refinement yielded the final models. The stereochemical quality of the final structures was verified using the program *PROCHECK* [47]. Statistics of the data collection and refinement are shown in Table 2. The structural data are available in the Protein Data Bank under the accession numbers 3VX0, 3VX1.

**Table 2:** Data-collection and refinement statistics.

Protein	Native	Gd derivative
No. of ligands		
NAG	1	1
Ca ion	1	1
Gd ion		6
Space group	*P*2_1_2_1_2_1_	*P*2_1_2_1_2_1_
Unit-cell parameter		
*a* (Å)	50.37	48.52
*b* (Å)	66.68	65.62
*c* (Å)	131.55	130.24
Wavelength (Å)	1.54	1.000
Resolution range (Å)	20–2.2 (2.28–2.20)	20–1.50 (1.55–1.50)
No. of unique reflections	23047 (2192)	66313 (6332)
Redundancy	6.6 (6.3)	5.7 (5.4)
Completeness (%)	98.6 (96.1)	98.5 (95.3)
*R*_merge_ † (%)	8.3 (33.6)	10.6 (54.1)
<*I*/*σ*(*I*)>	9.4 (5.6)	7.7 (3.6)
mosaicity (°)	0.77–0.86	0.46–0.65
Refinement		
Resolution range (Å)	20–2.2	20–1.50
*R*_cryst_/*R*_free_ §	20.8/24.6	19.2/19.9
No. of molecules in ASU	1	1
Rms deviation		
Bond length (Å)	0.009	0.009
Bond angle (°)	1.5	1.5
PDB code	3vx1	3vx0

Values in parentheses are for the outermost shell.

† *R*_merge_ = ∑*_hkl_* ∑*_i_* |*I_i_*(*hkl*)−<*I*(*hkl*)>|/∑*_hkl_* ∑*_i_ I_i_*(*hkl*), where *I_i_*(*hkl*) is the *i*th observation of reflection *hkl* and <*I*(*hkl*)> is the weighted average intensity for all observations *i* of reflection *hkl*.

§ *R*_cryst_ = ∑*_hkl_* ||*F*_obs_|−|*F*_calc_||/∑*_hkl_* |*F*_obs_|, where |*F*_obs_| and |*F*_calc_| are the observed and calculated structure-factor amplitudes, respectively. *R*_free_ was calculated with 5% of the reflections chosen at random and omitted from refinement.

### Dynamic light-scattering study

Ao α-amylase was examined by dynamic light-scattering experiment using a DynaPro MS/X (Protein Solutions) instrument at a protein concentration of 0.4 mg/mL in 50 m*M* MES-NaOH (pH 5.8) containing 0–2.0 m*M* GdCl_3_. Several measurements were taken at 293 K and analyzed using the program *DYNAMICS* v.5.26.60 (Protein Solutions). The average values of two measurements of mass at each GdCl_3_ concentration are shown in Table 1.

### CD spectra

Circular dichroism (CD) spectra of Ao α-amylase were recorded on a Jasco J-725 spectropolarimeter (Jasco Co., Japan). Near-UV CD spectra in the wavelength range from 250 to 320 nm were scanned 16 times at a scan rate of 20 nm/min, using a time constant of 0.25 sec. The light-path length of the cell was 10 mm in the near-UV region. For the measurements at 25°C, the protein concentrations were 0.75 mg/mL in 50 m*M* MES-NaOH (pH 5.8) with 0.04 m*M* or 0.2 m*M* GdCl_3_.
